# Trajectories of Vital Signs and Risk of In-Hospital Cardiac Arrest

**DOI:** 10.3389/fmed.2021.800943

**Published:** 2022-01-03

**Authors:** Chu-Lin Tsai, Tsung-Chien Lu, Chih-Hung Wang, Cheng-Chung Fang, Wen-Jone Chen, Chien-Hua Huang

**Affiliations:** ^1^Department of Emergency Medicine, National Taiwan University Hospital, Taipei, Taiwan; ^2^Department of Emergency Medicine, College of Medicine, National Taiwan University, Taipei, Taiwan

**Keywords:** cardiac arrest, vital sign, group-based trajectory modeling, in-hospital cardiac arrest, longitudinal modeling, emergency department (ED)

## Abstract

**Background:** Little is known about the trajectories of vital signs prior to in-hospital cardiac arrest (IHCA), which could explain the heterogeneous processes preceding this event. We aimed to identify clinically relevant subphenotypes at high risk of IHCA in the emergency department (ED).

**Methods:** This retrospective cohort study used electronic clinical warehouse data from a tertiary medical center. We retrieved data from 733,398 ED visits over a 7-year period. We selected one ED visit per person and retrieved patient demographics, triage data, vital signs (systolic blood pressure [SBP], heart rate [HR], body temperature, respiratory rate, oxygen saturation), selected laboratory markers, and IHCA status. Group-based trajectory modeling was performed.

**Results:** There were 37,697 adult ED patients with a total of 1,507,121 data points across all vital-sign categories. Three to four trajectory groups per vital-sign category were identified, and the following five trajectory groups were associated with a higher rate of IHCA: low and fluctuating SBP, high and fluctuating HR, persistent hypothermia, recurring tachypnea, and low and fluctuating oxygen saturation. The IHCA-prone trajectory group was associated with a higher triage level and a higher mortality rate, compared to other trajectory groups. Except for the persistent hypothermia group, the other four trajectory groups were more likely to have higher levels of C-reactive protein, lactic acid, cardiac troponin I, and D-dimer. Multivariable analysis revealed that hypothermia (adjusted odds ratio [aOR], 2.20; 95% confidence interval [95%CI], 1.35–3.57) and recurring tachypnea (aOR 2.44; 95%CI, 1.24–4.79) were independently associated with IHCA.

**Conclusions:** We identified five novel vital-sign sub-phenotypes associated with a higher likelihood of IHCA, with distinct patterns in clinical course and laboratory markers. A better understanding of the pre-IHCA vital-sign trajectories may help with the early identification of deteriorating patients.

## Introduction

In-hospital cardiac arrest (IHCA) is a major problem in the hospital and is associated with high morbidity and mortality worldwide (1). In the United States, the incidence of adult treated IHCA was about 10 per 1,000 hospital admissions (~290,000 patients per year), about 10% of which occurred in the emergency department (ED) (2, 3). Only about 25% of the IHCA patients survived to hospital discharge, and among them, 85% were discharged with a favorable neurological outcome (1).

Despite the catastrophic nature of IHCA, little is known about the trajectories of vital signs prior to IHCA. Early recognition and prevention have been added as the first link in the Chain of Survival for IHCA (4). Understanding the pre-IHCA physiological derangements, particularly longitudinal dynamic changes, would help clinicians recognize the patterns and the heterogeneous processes preceding the devastating event. Previous studies have utilized a snapshot of vital-sign data to predict IHCA (5, 6). In clinical practice, intermittently measured snapshot vital-sign data sometimes cause confusion as to appropriate responses, especially when no prior data are compared or are too late for interventions (7). Other studies have created summary measures for longitudinal vital-sign data (8), or have monitored early warning summary scores over time (9). As such, information is somewhat lost in terms of the dynamic changes of each vital sign over time, which may be more intuitive and clinically useful in phenotyping and prognosticating patients with IHCA. Group-based trajectory modeling (GBTM) is an unsupervised modeling technique to identify hidden subpopulations comprising similar individuals (10). To the best of our knowledge, no previous study has examined the latent trajectories of vital signs in patients with IHCA. Understanding the vital-sign change patterns prior to IHCA may gain lead time for appropriate interventions.

In this study, we aimed to identify clinically relevant sub-phenotypes at high risk of IHCA in the ED using longitudinal vital-sign data. We hypothesized that certain trajectory groups would have a higher likelihood of IHCA, with distinct patterns in clinical course and laboratory findings.

## Methods

### Study Design and Setting

We conducted a retrospective cohort study using data from the integrated Medical Database (iMD) of the National Taiwan University Hospital (NTUH). This database serves as a central clinical data warehouse for all electronic health records in the healthcare system (the main hospital and six branch hospitals), including inpatient, outpatient, and ED records. The electronic database houses a variety of information, including demographics, diagnosis, treatment, imaging, laboratory, prescription, nursing, billing, and administrative data. The database is maintained and updated by dedicated research personnel and has been used for clinical research studies (11, 12).

For the current study, we retrieved 7 years of de-identified iMD data from the NTUH main hospital between January 1, 2009 and December 31, 2015. The NTUH main hospital is a tertiary academic medical center with approximately 2,400 beds and 100,000 ED visits per year. The ED also manages an observation unit (EDOU), which is staffed by ED physicians. This study was approved by the NTUH Institutional Review Board, which waived the requirement for patient informed consent.

### Study Population

**Figure 1:**
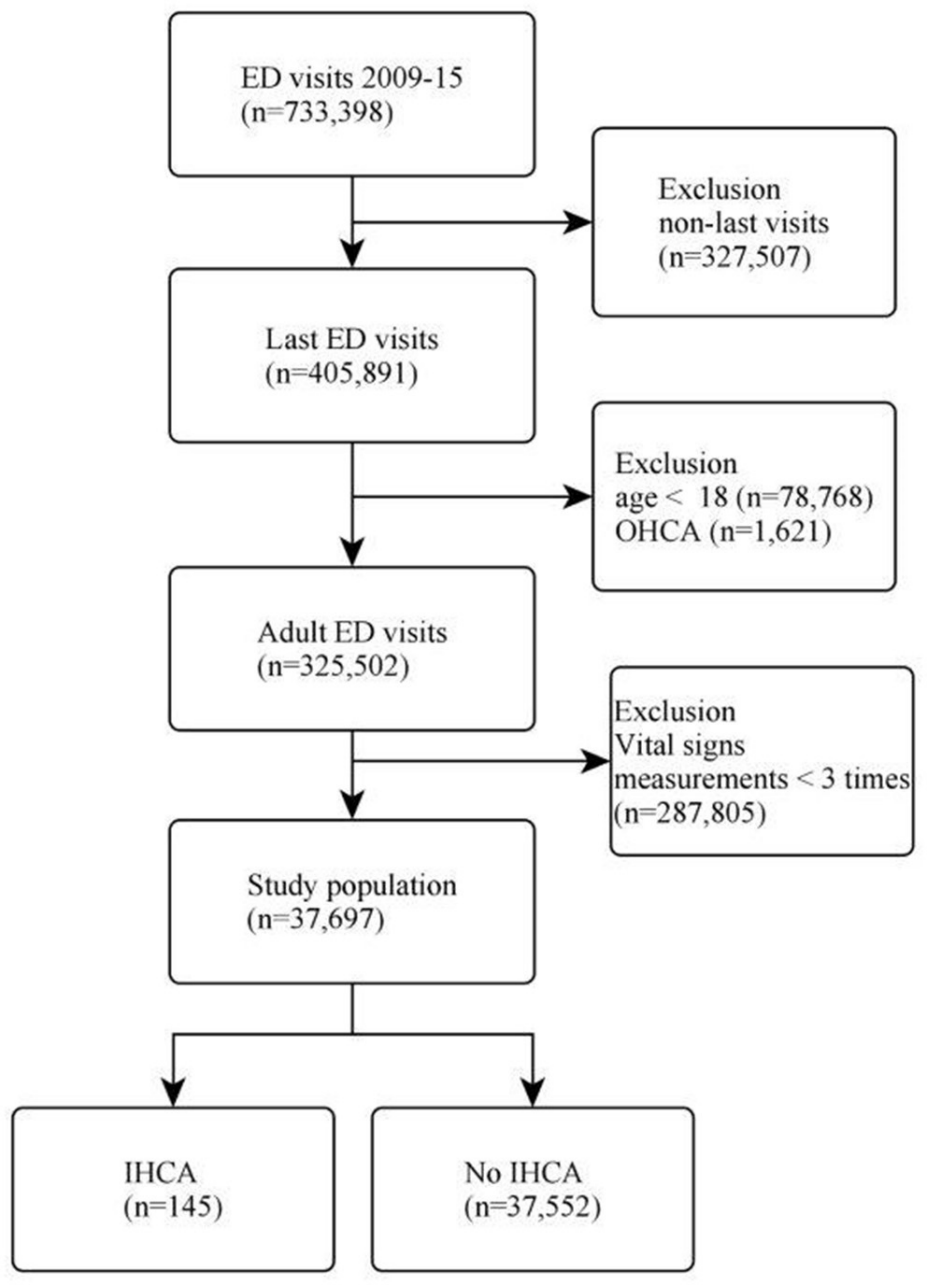
Flow diagram of the patient selection process. ED, emergency department; OHCA, out-of-hospital cardiac arrest; IHCA, in-hospital cardiac arrest.

We electronically extracted data from 733,398 ED visits over the 7-year period. For repeat visits, we selected the last visit per patient to maximize statistical power for the cardiac arrest analysis. If a patient had subsequent visits, it was much less likely that he/she suffered a cardiac arrest on a prior visit. We further excluded out-of-hospital cardiac arrests (OHCAs), patients aged less than 18 years, or those who had less than three vital-sign measurements. At least three measurements would ensure the stability of longitudinal analysis, and therefore many EDOU patients were included. The subject selection process is shown in Figure 1.

### Variables

Patient demographics and time-stamped clinical information in the ED were extracted, including chief complaint on ED presentation, mode of arrival, transfer status, serial vital sign measurements (systolic blood pressure [SBP], heart rate [HR], body temperature [BT], respiratory rate [RR], and oxygen saturation [SpO_2_]). The vital-sign measurements were from hour 0 (at ED triage) to the last measurement available or hour 191 (the timing of the last IHCA event), whichever occurred earlier. The vital-sign data were split into 1-h blocks, and if multiple measurements occurred in the 1-h block, the average value was used. The quick Sepsis-related Organ Failure Assessment (qSOFA) score at ED triage was calculated (13). Selected laboratory test results were also retrieved, including C-reactive protein (CRP), lactic acid, cardiac Troponin I (cTnI), and D-dimer. The ED-based IHCA was identified via a cardiopulmonary resuscitation (CPR) code (i.e., treated cardiac arrest). All laboratory results were the earliest available data in the ED. For IHCA patients, laboratory data were restricted to those obtained prior to CPR.

We also electronically extracted the five-level computerized Taiwan triage and acuity scale (TTAS) that contains information on a total of 179 structured chief complaints. The chief complaints included OHCA, which was used to identify the OHCA population. Based on the computerized algorithms, the TTAS classifies patients in the following order of acuity: level 1, resuscitation; level 2, emergent; level 3, urgent; level 4, less urgent; and level 5, non-urgent. The TTAS was adapted from the Canadian Triage and Acuity Scale and has been validated against hospitalization, length of ED stay, and resource utilization (14). To study the possible causes of IHCA, the primary fields of ED discharge diagnosis codes were grouped into clinically meaningful categories using the Clinical Classification Software (CCS) (15).

The data extractors were hospital information technology engineers who were blinded to the study hypothesis. The data underwent electronic cleaning, and invalid data were set to missing values after periodic investigator meetings.

### Outcome Measures

The primary outcome measure, ED-based IHCA, was identified via a cardiopulmonary resuscitation (CPR) procedure code (i.e., treated cardiac arrest). Patients with do-not-resuscitate (DNR) status were not counted as treated cardiac arrests, according to consensus guidelines on reporting IHCA (16). The secondary outcome measure was mortality in the ED.

### Statistical Analysis

Summary statistics are presented as proportions (with 95% confidence intervals [CIs]), means (with standard deviations [SDs]), or medians (with interquartile ranges [IQRs]). Bivariate associations were examined using Student *t*-tests, Mann-Whitney tests, and chi-square tests, as appropriate. Patient characteristics, laboratory findings, mortality, and IHCA status were compared between the identified trajectory groups. We used available-case analysis for the laboratory analysis as not all patients had test results available.

Group-based trajectory modeling (GBTM) was performed to identify trajectory groups in each of the five vital-sign categories. GBTM is an explanatory modeling technique to identify hidden groups of individuals with similar trajectories for a particular variable of interest (17). This technique uses finite mixture modeling to identify clusters of longitudinal data (18). We tested models of two to six groups with the inclusion of constant, linear, quadratic, or cubic terms. The Bayesian information criterion was used to choose the optimal number and form of trajectories. GBTM was performed using the traj package in Stata software (StataCorp, College Station, TX, USA).

After the unsupervised identification of longitudinal trajectories, we used supervised multivariable logistic regression to examine the independent association between the trajectory group memberships and ED-based IHCA, controlling for age, sex, and triage levels. To internally validate the identified trajectory groups, we bootstrapped the model 100 times to obtain the bias-corrected confidence intervals. All odds ratios (ORs) and beta-coefficients are presented with 95% CIs. To test if the trajectory groups using the early data could also relate to the occurrence of IHCA, we conducted a sensitivity analysis by restricting vital-sign measurements from 0 to 24h.

All analyses were performed using Stata 16.0 software. All *P* values are two-sided, with *P* < 0.05 considered statistically significant.

## Results

Of 733,398 ED visits during the 7-year study period, 405,891 unique patient visits were included. After excluding patients aged less than 18 years or patients with out-of-hospital cardiac arrest, 325,502 adult visits were included in the analysis. We further excluded those who had vital signs measurements less than three times, leaving 37,697 patients in the analysis. The patient selection process is shown in Figure 1. There were 145 (0.4%) patients who developed IHCA in the ED.

Overall, the mean age of these patients was 63 years, and 46% were women (Table 1). Most patients arrived in the ED during the daytime or in the evening, and patients were evenly distributed across seasons. Most patients presented with fever, followed by dyspnea and abdominal pain, were triaged to level 3, and had a median qSOFA score of 0 (IQR, 0–1). The initial vital signs were generally acceptable, except for slightly faster HR and lower SpO_2_. The overall incidence of ED-based IHCA was 0.4%, with a median time to IHCA of 40 h. About 63% were admitted to the hospital, and 2% died in the ED. The median ED/EDOU length of stay was about 47 h. Among patients in whom laboratory tests were ordered, some laboratory abnormalities were observed (e.g., leukocytosis and elevated levels of CRP, lactic acid, cTnI, and D-dimer). The baseline clinical characteristics of the 145 IHCA patients are shown in the Supplementary Table 1. The most common discharge diagnoses/symptoms for ED/EDOU patients with IHCA were pneumonia, gastrointestinal hemorrhage, and fever (Supplementary Table 2). Of the IHCA patients, 122 (84%) were intubated and mechanically ventilated.

**Table 1:** Baseline clinical characteristics of emergency department patients.

**Variable**	***N*** **= 37,697**
Age, mean (SD), yr	62.9 (18.3)
Female sex, *n* (%)	17,258 (45.8)
Season, *n* (%)	
Spring (Mar. – May)	8,410 (22.3)
Summer (Jun. – Aug.)	10,430 (27.7)
Fall (Sep. – Nov.)	9,721 (25.8)
Winter (Dec. – Feb.)	9,136 (24.2)
Presenting time, *n* (%)	
7:00 am to 2:59 pm	17,071 (45.3)
3:00 pm to 10:59 pm	15,692 (41.6)
11:00 pm to 6:59 am	4,934 (13.1)
Most common chief complaint, *n* (%)	
Fever	5,703 (15.3)
Dyspnea	5,409 (14.5)
Abdominal pain	4,993 (13.4)
Triage level, *n* (%)	
1	2,013 (5.3)
2	13,022 (34.5)
3	21,765 (57.7)
4	827 (2.2)
5	70 (0.2)
qSOFA at triage, median (IQR)	0 (0–1)
Vital sign at triage	
Systolic blood pressure, mean (SD), mmHg	136.3 (31.1)
Heart rate, mean (SD), beats per min	96.5 (22.1)
Body temperature, mean (SD), °C	37.2 (1.1)
Respiratory rate, mean (SD), breaths per min	19.3 (2.9)
Oxygen saturation, median (IQR), %	97 (95–98)
IHCA, *n* (%)	145 (0.4)
Time to IHCA, median (IQR), hr	39.6 (9.2–83.4)
Hospital admission, *n* (%)	23,614 (62.6)
ED mortality, *n* (%)	805 (2.1)
ED/EDOU length of stay, median (IQR), hr	46.6 (22.4–81.6)
Selected laboratory results	
WBC count (K/μL) (<10 K/μL)^a^	9.4 (6.7–13.2)
C–reactive protein (mg/dL) (<1 mg/dL)^b^	5.0 (1.3–12.0)
Lactic acid (mmole/L) (<2 mmole/L)^c^	2.0 (1.3–3.2)
Cardiac troponin I (ng/mL) (<0.05 ng/mL)^d^	0.0 (0.0–0.1)
D–dimer (mg/L) (<0.5 mg/L)^e^	1.8 (0.6–5.0)

a *Available in 29,007 patients*.

b *Available in 5,884 patients*.

c *Available in 8,556 patients*.

d *Available in 11,602 patients*.

e *Available in 2,776 patients. SD, standard deviation; qSOFA, quick Sepsis- related Organ Failure Assessment; IQR, interquartile range; IHCA, in-hospital cardiac arrest; ED, emergency department; EDOU, emergency department observation unit; WBC, white blood cell*.

**Figure 2:**
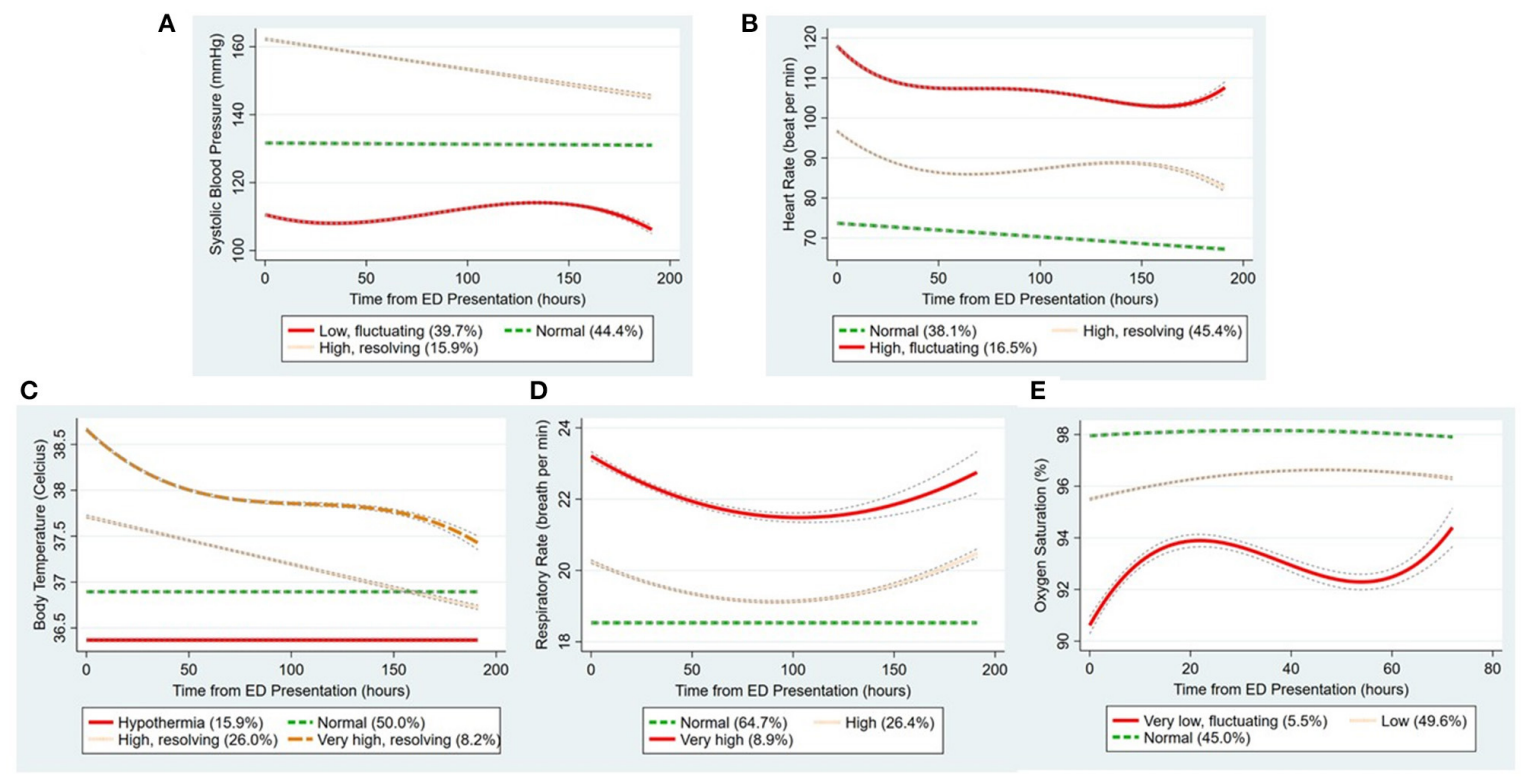
The trajectory groups identified by group-based trajectory modeling in each vital-sign category. **(A-E)** Indicate systolic blood pressure, heart rate, body temperature, respiratory rate, and oxygen saturation, respectively. The percentage in parenthesis denotes the proportion of patients in that trajectory group. The lines around the trajectory show the confidence intervals. ED, emergency department.

The 37,697 patients contributed to a total of 1,507,121 data points across all vital-sign categories. Each patient was measured multiple times, with a median measurement of 7 times (IQR, 4–13 times). Figure 2 depicts the trajectory groups in each vital-sign category. Three to four trajectory groups per vital sign were identified by the trajectory modeling. For example, in the SBP category, three distinct trajectory groups were identified, representing “normal” (44% of patients), “high, resolving” (16%), and “low, fluctuating” (40%) SBP over time. Similarly, three trajectory groups were identified for the longitudinal HR data. Notably, four trajectory groups were identified in the body temperature category, representing “hypothermia” (16%), “normal” (50%), “high, resolving” (26%), “very high, resolving” (8%) groups. For the longitudinal RR and SpO_2_ data, three trajectory groups were identified for each vital-sign category. The detailed summary measurements (initial value, mean, minimum, maximum, and standard deviation) for each vital-sign category is shown in Supplementary Tables 3, 4.

Tables 2, 3 show the clinical characteristics and outcomes of the trajectory groups in each vital-sign category. Across all vital-sign categories, the following five trajectory groups were associated with a higher rate of IHCA: low and fluctuating SBP, high and fluctuating HR, persistent hypothermia, initially-resolving but recurring tachypnea, and low and fluctuating oxygen saturation. Within each vital-sign category, the IHCA-prone trajectory group was associated with a higher triage level and a higher mortality rate, compared to other trajectory groups. Except for the persistent hypothermia group, the other four IHCA-prone trajectory groups were more likely to have a higher qSOFA score and higher levels of CRP, lactic acid, cTnI, and D-dimer, compared with other trajectory groups in their corresponding vital-sign category.

**Table 2:** Patient characteristics and clinical outcomes by the trajectory group (systolic blood pressure and heart rate).

**Variable**	**SBP group**				**HR group**			
	**Low, fluctuating**	**Normal**	**High, resolving**	***P*** **value**	**Normal**	**High, resolving**	**Very high, fluctuating**	***P*** **value**
	**(***n =*** 14,974)**	**(***n =*** 16,822)**	**(***n =*** 5,901)**		**(***n =*** 14,344)**	**(***n =*** 17,246)**	**(***n =*** 6,107)**	
Age, mean (SD), yr	57.7 (19.1)	65.4 (17.3)	69.0 (15.1)	<0.001	63.7 (18.2)	62.4 (18.5)	62.2 (17.8)	<0.001
Female sex, *n* (%)	7,223 (48.2)	7,263 (43.2)	2,772 (47.0)	<0.001	6,743 (47.0)	7,914 (45.9)	2,601 (42.6)	<0.001
Triage, *n* (%)				<0.001				<0.001
1	1,055 (7.0)	717 (4.3)	241 (4.1)		479 (3.3)	874 (5.1)	660 (10.8)	
2	4,860 (32.5)	5,330 (31.7)	2,832 (48.0)		4,793 (33.4)	5,708 (33.1)	2,521 (41.3)	
3	8,609 (57.5)	10,364 (61.6)	2,792 (47.3)		8,665 (60.4)	10,257 (59.5)	2,843 (46.6)	
4	417 (2.8)	377 (2.2)	33 (0.6)		377 (2.6)	371 (2.2)	79 (1.3)	
5	33 (0.2)	34 (0.2)	3 (0.1)		30 (0.2)	36 (0.2)	4 (0.1)	
Most common chief complaint, *n* (%)	Fever				Fever			
	2751 (18.6)	2526 (15.2)	426 (7.3)	<0.001	1244 (8.8)	3257 (19.1)	1202 (20.0)	<0.001
qSOFA, median (IQR)	0 (0–1)	0 (0–1)	0 (0–0)	<0.001	0 (0–0)	0 (0–1)	1 (0–1)	<0.001
CRP, median (IQR), mg/dL^a^ (<1 mg/dL)	5.7 (1.7–12.5)	4.9 (1.3–11.8)	2.9 (0.7–9.1)	<0.001	2.1 (0.5–7.1)	5.6 (1.8–12.2)	9.1 (3.7–16.3)	<0.001
Lactic acid, median (IQR), mmole/L^b^ (<2 mmole/L)	2.2 (1.4–3.7)	1.9 (1.3–2.9)	1.6 (1.1–2.5)	<0.001	1.6 (1.1–2.4)	1.9 (1.3–3.1)	2.6 (1.6–4.6)	<0.001
cTnI, median (IQR), ng/mL^c^ (<0.05ng/mL)	0.03 (0.01–0.08)	0.02 (0.01–0.06)	0.03 (0.01–0.07)	<0.001	0.02 (0.01–0.05)	0.03 (0.01–0.07)	0.04 (0.01–0.09)	<0.001
D–dimer, median (IQR), mg/L^d^ (<0.5 mg/L)	2.2 (0.8–5.9)	1.8 (0.6–4.9)	1.2 (0.5–3.5)	<0.001	0.9 (0.3–2.4)	2.1 (0.8–5.4)	3.4 (1.6–8.9)	<0.001
IHCA, *n* (%)	81 (0.5)	45 (0.3)	19 (0.3)	<0.001	36 (0.3)	59 (0.3)	50 (0.8)	<0.001
ED mortality, *n* (%)	543 (3.6)	218 (1.3)	44 (0.7)	<0.001	100 (0.7)	274 (1.6)	431 (7.1)	<0.001

a *Available in 5,884 patients*.

b *Available in 8,556 patients*.

c *Available in 11,602 patients*.

d *Available in 2,776 patients. SBP, systolic blood pressure; HR, heart rate; SD, standard deviation; qSOFA, quick Sepsis–related Organ Failure Assessment; IQR, interquartile range; CRP, C–reactive protein; cTnI, cardiac troponin I; IHCA, in–hospital cardiac arrest; ED, emergency department*.

**Table 3:** Patient characteristics and clinical outcomes by the trajectory group (body temperature, respiratory rate, and oxygen saturation).

**Variable**	**BT group**					**RR group**				**SpO_2_ group**			
	**Hypothermia**	**Normal**	**High, resolving**	**Very high**,	***P*** **value**	**Normal**	**High**	**Very high**	***P*** **value**	**Very low,**	**Low**	**Normal**	*P* **value**
	**(***n =*** 4,771)**	**(***n =*** 18,932)**	**(***n =*** 8,998)**	**resolving** **(***n =*** 2,810)**		**(***n =*** 15,642)**	**(***n =*** 5,526)**	**(***n =*** 1,954)**		**fluctuating** **(***n =*** 1,190)**	**(***n =*** 11,205)**	**(***n =*** 10,673)**	
Age, mean (SD), yr	67.3 (17.3)	62.9 (18.3)	61.9 (18.5)	57.5 (18.9)	<0.001	63.8 (17.7)	69.7 (16.5)	71.2 (16.0)	<0.001	72.2 (15.9)	68.7 (15.8)	62.0 (18.6)	<0.001
Female sex, *n* (%)	1,608 (33.7)	8,908 (47.1)	4,476 (49.7)	1,325 (47.2)	<0.001	6,899 (44.1)	2,290 (41.4)	792 (40.5)	<0.001	457 (38.4)	4,661 (42.6)	4,840 (45.4)	<0.001
Triage, *n* (%)					<0.001				<0.001				<0.001
1	329 (6.9)	762 (4.0)	368 (4.1)	136 (4.8)		600 (3.8)	684 (12.4)	618 (31.6)		557 (46.8)	648 (5.8)	676 (6.3)	
2	1,741 (36.5)	6,361 (33.6)	2,700 (30.0)	812 (28.9)		5,558 (35.5)	2,659 (48.1)	919 (47.0)		419 (35.2)	4,645 (41.5)	4,040 (37.9)	
3	2,553 (53.5)	11,238 (59.4)	5,785 (64.3)	1,836 (65.3)		8,969 (57.3)	2,129 (38.5)	409 (20.9)		208 (17.5)	5,690 (50.8)	5,606 (52.5)	
4	134 (2.8)	528 (2.8)	132 (1.5)	25 (0.9)		480 (3.1)	49 (0.9)	7 (0.4)		5 (0.4)	207 (1.9)	326 (3.1)	
5	14 (0.3)	43 (0.2)	13 (0.1)	1 (0.0)		35 (0.2)	5 (0.1)	1 (0.1)		1 (0.1)	15 (0.1)	25 (0.2)	
Most common chief complaint, n (%)	Fever					Dyspnea					Dyspnea		
	122 (2.6)	1,399 (7.5)	2,980 (33.4)	1,433 (51.7)	<0.001	1,612 (10.4)	2,170 (39.9)	1,140 (59.4)	<0.001	663 (55.2)	2,611 (23.3)	1,770 (16.4)	<0.001
qSOFA, median (IQR)	0 (0–1)	0 (0–1)	0 (0–1)	0 (0–1)	<0.001	0 (0–1)	1 (0–1)	1 (1–2)	<0.001	1 (1–2)	0 (0–1)	0 (0–1)	<0.001
CRP, median (IQR), mg/dL^a^ (<1 mg/dL)	2.6 (0.5–7.5)	3.6 (0.8–9.1)	7.1 (2.4–14.2)	8.4 (3.3–16.7)	<0.001	5.4 (1.5–12.3)	7.0 (2.1–15.0)	8.9 (4.1–16.0)	<0.001	8.5 (4.4–15.6)	7.1 (2.2–14.4)	4.6 (1.2–11.7)	<0.001
Lactic acid, median (IQR), mmole/L^b^ (<2 mmole/L)	2.1 (1.4–3.8)	1.9 (1.2–3.0)	1.9 (1.3–3.0)	2.1 (1.4–3.4)	<0.001	1.9 (1.3–3.0)	2.2 (1.4–4.0)	2.8 (1.7–4.9)	<0.001	3.1 (1.8–5.5)	2.0 (1.5–3.3)	2.0 (1.3–3.3)	<0.001
cTnI, median (IQR), ng/mL^c^ (<0.05ng/mL)	0.03 (0.01–0.08)	0.03 (0.01–0.06)	0.03 (0.01–0.07)	0.03 (0.01–0.08)	<0.001	0.02 (0.01–0.06)	0.04 (0.02–0.10)	0.05 (0.02–0.13)	<0.001	0.05 (0.02–0.13)	0.03 (0.01–0.08)	0.03 (0.01–0.07)	<0.001
D–dimer, median (IQR), mg/L^d^ (<0.5 mg/L)	1.8 (0.6–5.8)	1.6 (0.6–4.3)	2.8 (1.1–6.6)	3.0 (1.4–7.8)	<0.001	1.7 (0.6–4.6)	3.0 (1.1–7.5)	3.2 (1.6–8.7)	<0.001	4.1 (1.8–10.5)	2.3 (0.9–6.5)	1.8 (0.6–5.0)	<0.001
IHCA, n (%)	38 (0.8)	48 (0.3)	23 (0.3)	17 (0.6)	<0.001	48 (0.3)	53 (1.0)	23 (1.2)	<0.001	18 (1.5)	63 (0.6)	41 (0.4)	<0.001
ED mortality, n (%)	231 (4.8)	254 (1.3)	160 (1.8)	118 (4.2)	<0.001	105 (0.7)	235 (4.3)	435 (22.3)	<0.001	344 (28.9)	272 (2.4)	147 (1.4)	<0.001

a *Available in 5,884 patients*.

b *Available in 8,556 patients*.

c *Available in 11,602 patients*.

d *Available in 2,776 patients. BT, body temperature; RR, respiratory rate; SpO_2_, oxygen saturation; SD, standard deviation; qSOFA, quick Sepsis- related Organ Failure Assessment; IQR, interquartile range; CRP, C-reactive protein; cTnI, cardiac troponin I; IHCA, in-hospital cardiac arrest; ED, emergency department*.

The bootstrapped multivariable analysis showed that hypothermia (vs. normothermia) and higher RR groups (high/very high, recurring tachypnea vs. normal RR group) were independently associated with IHCA, adjusting for age, sex, triage levels, and other trajectory groups (Table 4). For example, hypothermia was independently associated with a 2.2-fold increased risk of IHCA (95% CI, 1.35–3.57).

**Table 4:** Bootstrapped multivariable model of trajectory groups associated with emergency department in-hospital cardiac arrest.

**Variable**	**Adjusted odds ratio**	**95% confidence interval**	***P*** **value**
BT trajectory group			
Hypothermia	**2.20**	1.35–3.57	0.001
Normothermia (reference)	1.00		
High, resolving	0.95	0.50–1.80	0.880
Very high, resolving	1.60	0.85–2.99	0.142
RR trajectory group			
Normal (reference)	1.00		
High, recurring	**2.51**	1.54–4.09	<0.001
Very high, recurring	**2.44**	1.24–4.79	0.010

*Significant odds ratios are highlighted in bold. BT, body temperature; RR, respiratory rate. Model adjusted for age, sex, triage level, and all five trajectory groups (systolic blood pressure, heart rate, body temperature, respiratory rate, and oxygen saturation). Statistically non-significant predictors are not shown*.

In the sensitivity analysis by restricting the GBTM analysis to hours 0–24, the early trajectories were quite similar to the entire trajectories, except that some fever trajectories had not fully resolved to the normal level and that recurring tachypnea had not fully appeared (Supplementary Figure 1). The aforementioned five trajectories using early data were still associated with IHCA (*P* ≤ 0.001), expect for the high RR groups.

## Discussion

In this study of 37,697 patients comprising >1 million longitudinal data points, we discovered five distinct vital-sign trajectory groups associated with a higher rate of IHCA. In contrast to other four trajectory groups, the hypothermia group appeared to have a unique pattern of suppressed laboratory markers findings. The internally validated multivariable analysis suggested hypothermia and recurring tachypnea were independently associated with IHCA.

A common theme across the five IHCA-prone vital-sign trajectory groups was the initial deviation from the norm, followed by a persistent deviation without resolution. In general, within each vital-sign category, those whose vital signs resolved to near normal (e.g., initially high but resolving SBP or HR) tended to have better outcomes, compared with those with persistently deviating vital signs. The degree to which the vitals deviated and fluctuated also differed by the vital-sign category. For instance, the fluctuation of SBP and HR seemed smaller, compared to that of RR and SpO_2_. This smaller longitudinal variation might also implicate that hypotension and tachycardia in the early phase of ED stay could predict worse outcomes. Indeed, studies have shown the “shock index” at triage, defined as HR divided by SBP, predicted hospital admission and inpatient mortality among ED patients (19). The higher levels of lactic acid, cTnI, and D-dimer may reflect the combination of various forms of shock (e.g., septic or cardiogenic) in the groups of low/fluctuating SBP and high/fluctuating HR. On the other hand, high blood pressure in the ED is common but does not seem to be associated with subsequent adverse events (20).

The hypothermia trajectory group appeared to have a relatively flat temperature course and relatively low levels of inflammatory markers. Consistent with previous studies (21–23), we found that hypothermia was associated with a higher rate of mortality and extended this association to include IHCA. Hypothermia is known to be arrhythmogenic and could be related to sepsis, both of which may have contributed to IHCA in our study. The hypothermia group demonstrated a “hypoinflammatory” state as evidenced by a low qSOFA score and a lower level of CRP. These patients were older and were probably immunosuppressed, and therefore their immune response was insufficient to generate fever. A previous study also showed that hypothermia in sepsis patients was associated with persistent lymphopenia, a sepsis-induced immunosuppression (24). Our study further demonstrated that the hypoinflammatory state usually persisted, at least in the ED, and predicted worse outcomes. On the other hand, a study has reported that hyperthermic patients in the ED received more antibiotic therapy, and thus had lower mortality compared with normothermic patients (25).

Multivariable analysis suggested that, besides hypothermia, an initially resolving but recurring tachypnea was independently associated with IHCA. Many IHCA events are caused by respiratory failure, such as acidosis and pneumonia, and cardiac causes (26). Prior to IHCA, tachypnea is often present as a compensatory response to shock-induced metabolic acidosis. A previous ward study also reported RR was the most important vital sign in predicting critical events (8). In our study, the degree of fluctuation of RR and SpO_2_ over time was quite large, indicating dynamic changes between treatment response and failure and a strong terminal deviation from the norm. Among various diseases, pneumonia may particularly alter the longitudinal patterns of RR and SpO_2_. A previous study has shown that deteriorating pneumonia demonstrated rapidly-worsening respiratory failure, with high RR and low SpO_2_, but only minor changes in other vital signs (27). Our study also suggested that, unlike the evolution of SBP and HR, the changes in RR and SpO_2_ could be drastic just prior to IHCA. As shown in the sensitivity analysis, the early RR trajectory was not reliable to detect imminent IHCAs. Recognizing these late changing respiratory trajectories, along with various important clinical and social factors, might prompt an early discussion of the risks and benefits of airway interventions or do-not-resuscitate orders if not already available.

This study has some potential limitations. First, this was a single-center study at a tertiary medical center, and our findings may not be generalizable to hospitals of different settings. Second, our study population was restricted to those who underwent at least three measurements of vital signs to ensure the stability of statistical analysis. As such, less ill patients were excluded, and the findings may not be applied to them. At the other end, our study population included EDOU patients potentially awaiting an inpatient bed, and our results may be potentially useful for EDOU and hospitalized patients. Third, we did not control for the medication effects (e.g., inotropes and antipyretics), which may have altered the trajectories. However, this rendered our results more reflective of a real-world situation. Finally, although we performed bootstrapping to internally validate our results, the identified trajectories and their relationships with IHCA still need to be externally validated in future large studies.

## Conclusions

In summary, in this large study of 37,697 patients with about 1.5 million longitudinal data points, we identified five novel vital-sign sub-phenotypes associated with a higher likelihood of IHCA, with distinct patterns in clinical course and laboratory markers. A better understanding of the pre-IHCA vital-sign trajectories may help with the early identification of deteriorating patients and has potential implications for personalized prevention of IHCA.

## Data Availability Statement

The original contributions presented in the study are included in the article/Supplementary Material, further inquiries can be directed to the corresponding author/s.

## Ethics Statement

The studies involving human participants were reviewed and approved by NTUH Institutional Review Board. Written informed consent for participation was not required for this study in accordance with the national legislation and the institutional requirements.

## Author Contributions

C-LT and T-CL: study concept and design, acquisition of data, and statistical analysis. C-LT: first drafting of the manuscript, had access to all the data in the study, takes responsibility for the integrity of the data, and the accuracy of the data analysis. C-LT and C-HH: obtained funding and study supervision. All authors: analysis and interpretation of data, critical revision of the manuscript for important intellectual content, administrative, technical, and material support.

## Funding

This project was supported by grants from the National Taiwan University Hospital, the Far Eastern Memorial Hospital, and the Ministry of Science and Technology grants (109-2634-F002-041 and 110-2634-F-002-046).

## Conflict of Interest

The authors declare that the research was conducted in the absence of any commercial or financial relationships that could be construed as a potential conflict of interest.

## Publisher's Note

All claims expressed in this article are solely those of the authors and do not necessarily represent those of their affiliated organizations, or those of the publisher, the editors and the reviewers. Any product that may be evaluated in this article, or claim that may be made by its manufacturer, is not guaranteed or endorsed by the publisher.

## Supplementary Material

Supplementary Figure 1 The trajectory groups identified by group-based trajectory modeling in each vital-sign category within 24 hours. **(A-E)** Indicate systolic blood pressure, heart rate, body temperature, respiratory rate, and oxygen saturation, respectively.

Supplementary Table 1 Baseline clinical characteristics of emergency department patients with in-hospital cardiac arrest.

Supplementary Table 2 Most common discharge diagnoses of emergency department patients with in-hospital cardiac arrest.

Supplementary Table 3 The detailed summary measurements (initial value, mean, minimum, maximum, and standard deviation) for each vital-sign category.

Supplementary Table 4 The detailed summary measurements (initial value, mean, minimum, maximum, and standard deviation) for each vital-sign category.
