# Supplementary figures and images for: Trajectories of Vital Signs and Risk of In-Hospital Cardiac Arrest
Source: Front Med (Lausanne). 2022 Jan 3;8:800943. doi: 10.3389/fmed.2021.800943 (PMC8761796; doi:10.3389/fmed.2021.800943)

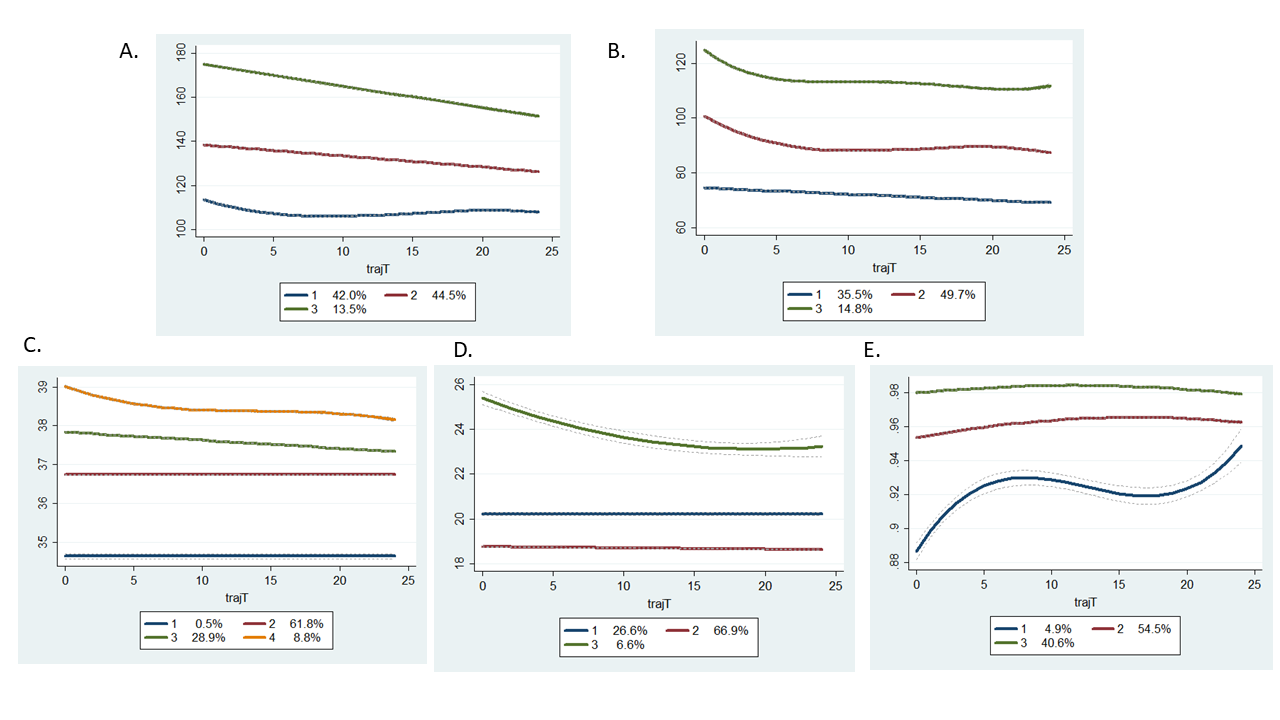

Supplement: Supplementary Figure 1 — The trajectory groups identified by group-based trajectory modeling in each vital-sign category within 24 hours. (A-E) Indicate systolic blood pressure, heart rate, body temperature, respiratory rate, and oxygen saturation, respectively. [file Image_1.TIF]
