# Supplementary material for: Trajectories of Vital Signs and Risk of In-Hospital Cardiac Arrest
Source: Front Med (Lausanne). 2022 Jan 3;8:800943. doi: 10.3389/fmed.2021.800943 (PMC8761796; doi:10.3389/fmed.2021.800943)
Supplement: Supplementary Table 1 — Baseline clinical characteristics of emergency department patients with in-hospital cardiac arrest. [file Table_1.DOCX]

**Online Supplementary eTable 1**. Baseline clinical characteristics of emergency department patients with in-hospital cardiac arrest.

| **Variable** | **N=145** |
| --- | --- |
| Age, mean (SD), yr | 67.7 (15.3) |
| Female sex, n (%) | 61 (42.1) |
| Season, n (%) |  |
| Spring (Mar. – May) | 30 (20.7) |
| Summer (Jun. – Aug.) | 43 (29.7) |
| Fall (Sep. – Nov.) | 34 (23.5) |
| Winter (Dec. – Feb.) | 38 (26.2) |
| Presenting Time, n (%) |  |
| 7:00 am to 2:59 pm | 77 (53.1) |
| 3:00 pm to 10:59 pm | 42 (29.0) |
| 11:00 pm to 6:59 am | 26 (17.9) |
| Most common chief complaint, n (%) |  |
| Dyspnea | 27 (18.6) |
| Fever | 17 (11.7) |
| Abdominal pain | 9 (6.2) |
| Triage level, n (%) |  |
| 1 | 19 (13.1) |
| 2 | 57 (39.3) |
| 3 | 67 (46.2) |
| 4 | 2 (1.4) |
| qSOFA at triage, median (IQR) | 1 (0-1) |
| Vital sign at triage |  |
| Systolic blood pressure, mean (SD), mmHg | 125.0 (35.1) |
| Heart rate, mean (SD), beats per min | 99.1 (24.8) |
| Body temperature, mean (SD), °C | 36.9 (1.2) |
| Respiratory rate, mean (SD), breaths per min | 21.0 (4.3) |
| Oxygen saturation, median (IQR), % | 96 (94-98) |
| Time to IHCA, median (IQR), hr | 39.6 (9.2-83.4) |
| Intubated and mechanically ventilated, n (%) | 122 (84.1) |
| Hospital admission, n (%) | 64 (44.1) |
| ED mortality, n (%) | 81 (55.9) |
| ED/EDOU length of stay, median (IQR), hr | 40.4 (11.2-89.2) |

Abbreviations: SD = standard deviation; qSOFA = quick Sepsis- related Organ Failure Assessment; IQR = interquartile range; IHCA = in-hospital cardiac arrest; ED = emergency department; EDOU = emergency department observation unit.
