# Supplementary material for: Trajectories of Vital Signs and Risk of In-Hospital Cardiac Arrest
Source: Front Med (Lausanne). 2022 Jan 3;8:800943. doi: 10.3389/fmed.2021.800943 (PMC8761796; doi:10.3389/fmed.2021.800943)
Supplement: Supplementary Table 2 — Most common discharge diagnoses of emergency department patients with in-hospital cardiac arrest. [file Table_2.DOCX]

**Online Supplementary eTable 2**. Most common discharge diagnoses of emergency department patients with in-hospital cardiac arrest.

|  | Overall N=145 |
| --- | --- |
| Discharge diagnosis | n (%) |
| Pneumonia | 16 (11.2) |
| Gastrointestinal hemorrhage | 11 (7.7) |
| Fever | 9 (6.3) |
| Chest pain | 7 (4.9) |
| Shock | 7 (4.9) |
| Cancer of liver and biliary tract | 6 (4.2) |
| Cancer of head and neck | 5 (3.5) |
| Chronic obstructive pulmonary disease | 5 (3.5) |
| Renal failure | 5 (3.5) |
