# Supplementary material for: Trajectories of Vital Signs and Risk of In-Hospital Cardiac Arrest
Source: Front Med (Lausanne). 2022 Jan 3;8:800943. doi: 10.3389/fmed.2021.800943 (PMC8761796; doi:10.3389/fmed.2021.800943)
Supplement: Supplementary Table 3 — The detailed summary measurements (initial value, mean, minimum, maximum, and standard deviation) for each vital-sign category. [file Table_3.docx]

**Online Supplementary Table 3**. The detailed summary measurements (initial value, mean, minimum, maximum, and standard deviation) for each vital-sign category.

| Variable |  | SBP Group (mmHg) |  |  | HR Group  (beats per min) |  |
| --- | --- | --- | --- | --- | --- | --- |
|  | Low, fluctuating (n= 14,974) | Normal (n= 16,822) | High, resolving (n= 5,901) | Normal (n= 14,344) | High, resolving  (n= 17,246) | Very high, fluctuating (n= 6,107) |
| Mean of personal initial value | 115.1 | 140.3 | 174.6 | 79.2 | 101.8 | 120.9 |
| Mean of personal mean over time | 109.3 | 132.1 | 161.3 | 72.3 | 90.8 | 111.9 |
| Mean of personal minimum over time | 93.7 | 111.9 | 136.1 | 62.5 | 76.6 | 92.7 |
| Mean of personal maximum over time | 129.0 | 155.4 | 188.3 | 86.2 | 108.6 | 131.1 |
| Mean of personal SD over time | 15.3 | 12.0 | 18.9 | 8.6 | 11.1 | 12.5 |

Abbreviations: SBP = systolic blood pressure; HR = heart rate; SD = standard deviation.
