# Supplementary material for: Trajectories of Vital Signs and Risk of In-Hospital Cardiac Arrest
Source: Front Med (Lausanne). 2022 Jan 3;8:800943. doi: 10.3389/fmed.2021.800943 (PMC8761796; doi:10.3389/fmed.2021.800943)
Supplement: Supplementary Table 4 — The detailed summary measurements (initial value, mean, minimum, maximum, and standard deviation) for each vital-sign category. [file Table_4.DOCX]

**Online Supplementary Table 4**. The detailed summary measurements (initial value, mean, minimum, maximum, and standard deviation) for each vital-sign category.

| Variable |  | BT Group ( °C) |  |  |  | RR Group (breaths per min) |  |  | SpO_2_ Group (%) |  |
| --- | --- | --- | --- | --- | --- | --- | --- | --- | --- | --- |
|  | Hypothermia (n= 4,771) | Normal (n= 18,932) | High, resolving  (n= 8,998) | Very high, resolving  (n= 2,810) | Normal  (n= 15,642) | High  (n= 5,526) | Very high  (n= 1,954) | Very low, fluctuating  (n=1,190) | Low  (n=11,205) | Normal  (n=10,673) |
| Mean of personal initial value | 36.3 | 37.0 | 38.0 | 38.8 | 18.5 | 21.6 | 24.5 | 89 | 95 | 98 |
| Mean of personal mean over time | 36.3 | 36.9 | 37.6 | 38.3 | 18.5 | 19.8 | 22.8 | 92 | 96 | 98 |
| Mean of personal minimum over time | 35.7 | 36.3 | 36.7 | 37.2 | 17.0 | 17.3 | 17.8 | 80 | 93 | 96 |
| Mean of personal maximum over time | 36.9 | 37.5 | 38.6 | 39.6 | 20.1 | 23.8 | 30.4 | 97 | 98 | 99 |
| Mean of personal SD over time | 0.4 | 0.4 | 0.6 | 0.8 | 1.1 | 2.1 | 4.4 | 7 | 2 | 1 |

Abbreviations: BT = body temperature; RR = respiratory rate; SpO_2_ = oxygen saturation; SD = standard deviation.
